# Correction to “Bimetallic Copper–Silver Catalysts for the Electrochemical Reduction of CO_2_ to Ethanol”

**DOI:** 10.1021/acsaem.3c02818

**Published:** 2023-11-30

**Authors:** Elisabeth Robens, Burkhard Hecker, Hans Kungl, Hermann Tempel, Rüdiger-A. Eichel

In our original paper, ref 19 had not yet been accepted, so it could not be fully cited. Now, the paper is published and can be cited correctly as follows:

(19) Hecker, B.; Robens, E.; Valencia, H. E.; Windmüller, A.; Muench, F.; Meledina, M. Tempel, H.; Kungl, H.; Mayer, J.; Eichel, R.-A. Effects of Synthesized Silver Nanoplate Structures on the Electrochemical Reduction of CO_2_. *J. Electrochem. Soc.***2023**, *170* (9), 096505. DOI: 10.1149/1945-7111/acf3a0.

